# eGFR slope as a surrogate endpoint for clinical study in early stage of chronic kidney disease: from The Japan Chronic Kidney Disease Database

**DOI:** 10.1007/s10157-023-02376-4

**Published:** 2023-07-19

**Authors:** Seiji Itano, Eiichiro Kanda, Hajime Nagasu, Masaomi Nangaku, Naoki Kashihara

**Affiliations:** 1 Department of Nephrology and Hypertension, Kawasaki Medical School, 577 Matsushima, Kurashiki, Okayama 701-0192 Japan; 2 Medical Science, Kawasaki Medical School, Kurashiki, Okayama Japan; 3 Division of Nephrology and Endocrinology, The University of Tokyo Graduate School of Medicine, Tokyo, Japan

**Keywords:** eGFR slope, Chronic kidney disease, Surrogate endpoint, End stage kidney disease

## Abstract

**Background:**

In clinical trials targeting early chronic kidney disease (CKD), eGFR slope has been proposed as a surrogate endpoint for predicting end-stage kidney disease (ESKD). However, it is unclear whether the eGFR slope serves as a surrogate endpoint for predicting long-term prognosis in Japanese early CKD populations.

**Methods:**

The data source was the J-CKD-Database, which contains real-world data on patients with CKD in Japan. eGFR slope was calculated from the eGFR of each period, 1-year (1-year slope), 2-year (2-year slope), and 3-year (3-year slope), for participants with a baseline eGFR ≥ 30 ml/min/1.73 m^2^. The outcome was ESKD (defined as dialysis initiation or incidence of CKD stage G5). The relationship between eGFR slope and the sub-distribution hazard ratio (SHR) of ESKD with death as a competing event was investigated using a Fine-Gray proportional hazard regression model.

**Results:**

The number of participants and mean observation periods were 7768/877 ± 491 days for 1-year slope, 6778/706 ± 346 days for 2-year slope, and 5219/495 ± 215 days for 3-year slope. As the eGFR slope decreased, a tendency toward a lower risk of ESKD was observed. Compared with the 1-year slope, there was a smaller variation in the slope values for the 2-year or 3-year slope and a greater decrease in the SHR; therefore, a calculation period of 2 or 3 years for the eGFR slope was considered appropriate.

**Conclusion:**

Even in Japanese patients with early stage CKD, a slower eGFR slope calculated from eGFR values over 2–3 years was associated with a decreased risk of ESKD.

**Supplementary Information:**

The online version contains supplementary material available at 10.1007/s10157-023-02376-4.

## Introduction

Chronic Kidney Disease (CKD) is a global problem affecting a growing number of patients and healthcare economics [1]. Patients with CKD are at high risk for death, cardiovascular disease (CVD), and end-stage kidney disease (ESKD) [2–4]. Clinical hard endpoints, such as death, CVD, and ESKD, have been established in clinical studies of CKD. However, these true endpoints occur infrequently, require large sample sizes, and long observation periods. This results in higher study costs and reduced feasibility, which are barriers to conducting clinical trials on kidney disease [5]. Therefore, surrogate endpoints for hard renal outcomes have been investigated.

In advanced CKD (G3b-G5 in the CKD staging), a 30–40% drop in eGFR or doubling of serum creatinine levels was established as a surrogate endpoint for ESKD by KDIGO (Kidney Disease Improving Global Outcomes) in 2017 [6]. These surrogate endpoints were also validated in Japanese populations and published as guidelines for clinical evaluation of chronic kidney disease [7]. In 2018, a scientific workshop sponsored by the National Kidney Foundation (NKF), US Food and Drug Administration (FDA), and European Medicines Agency (EMA) recommended a surrogate endpoint in early CKD [8, 9]. In this recommendation, the change in eGFR slope was evaluated as a surrogate endpoint, eGFR slope reduction 0.5–1.0 mL/min/1.73 m^2^/year is a cutoff value that predicts ESKD risk reduction. However, considering the non-negligible differences in eGFR at the time of dialysis initiation between Japan and other countries [10–13], it is necessary to determine whether the same values can be applied to Japanese patients.

Therefore, the purpose of this study was to examine the external validity of the surrogate endpoints recommended by the NKF-FDA-EMA workshop to be similarly applicable to Japanese CKD patients. We examined the association between the change in eGFR slope and ESKD using data from the J-CKD-Database (J-CKD-DB) [14], a real-world dataset of CKD patients in Japan.

## Materials and methods

### Data source and exclusion criteria

The data source was the J-CKD-DB database [14], which was designed to collect real-world data on CKD patients in domestic university hospitals. In brief, this database uses SS-MIX2 standardized storage to automatically extract data on CKD cases from electronic medical records. The criteria for registering CKD patients in J-CKD-DB are as follows: (1) age ≥ 18 years old and (2) proteinuria ≥ 1 + (dipstick test) and/or eGFR < 60 mL/min/1.73 m^2^, during the study period. In this study, we utilized the J-CKD-DB-Ex, an advanced version of the JCKDDB, which includes longitudinal data spanning multiple years.

We excluded participants who met the exclusion criteria from all patients registered in the database from 2014 to 2018. The exclusion criteria were as follows: (1) participants with less than two eGFR measurements, including the baseline measurement needed for eGFR slope calculation; (2) participants with no data available after the eGFR slope calculation period of 1 to 3 years; (3) participants with missing covariate data (any missing covariate data resulted in exclusion from the analysis); (4) participants without data on initiation of dialysis or death; and 5) participants with a baseline eGFR less than 30 mL/min/1.73m^2^. (This study aimed to establish surrogate endpoints for early CKD, the participants eGFR < 30 mL/min/1.73m^2^ were excluded as advanced CKD. Additionally, as an exploratory analysis, we also analyzed in a population with more advanced renal failure, eGFR between 20 and 30 mL/min/1.73 m^2^.)

### Definition of outcome and observation period

**Fig. 1:**
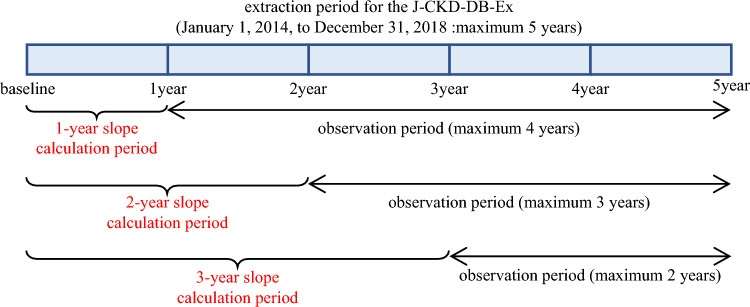
Calculation period and observation period for eGFR slope. The extraction period for J-CKD-DB-Ex was from January 1, 2014, to December 31, 2018, and data for up to five years were extracted. The 1-year slope was calculated using data from days 1 to 365 for participants with eGFR data for 366 days or more from baseline. The 2-year slope was calculated using data from days 1 to 730 for participants with eGFR data for 731 days or more from baseline. The 3-year slope was calculated using data from day 1 to day 1095 for participants with eGFR data for 1096 days or more from baseline. The observation period was defined as the period from the end of the eGFR slope calculation to the occurrence of the outcome or final eGFR measurement

The outcome was defined as ESKD. In the primary analysis, ESKD was defined as dialysis initiation. In the secondary analysis, ESKD was defined as the incidence of CKD stage G5 (eGFR < 15 mL/min/1.73 m^2^). The observation period was defined as the period from after the eGFR slope calculation to the occurrence of the outcome or final eGFR measurement (Fig. 1).

### eGFR slope

The eGFR values were calculated using the Japanese GFR estimation equation [15] based on serum creatinine values. The oldest eGFR measurement point for each case was used as the baseline. For participants who had undergone two or more eGFR measurements, including the baseline, during the calculation period, the eGFR slope was calculated using all eGFR values within the calculation period. The eGFR slope was calculated using the least-squares method and the mixed-effects model. The least squares method used the slope of the linear approximation model, which minimized the sum of the squared residuals between the eGFR value and the model, as the eGFR slope. The mixed-effects model used a linear mixed-effects model with random intercepts and slopes to estimate the slope for each individual. Three types of eGFR slopes were calculated using eGFR values from 1, 2, and 3 years after baseline, and were used as explanatory variables. Any participants that deviated from the range of the mean value ± 3SD were excluded from the analysis.

### Statistical analysis

For baseline information on the study participants, mean ± SD or median (interquartile range) was used to present continuous variables, while numbers and percentages were used for binary variables. For both the dialysis initiation and incidence of CKD stage G5, which compete with death events, multivariate analysis was performed using the Fine-Gray proportional hazard regression model. The hazard risk of outcome occurrence was estimated for each change in the eGFR slope. Subgroup analysis was performed for (1) baseline eGFR ≥ 45 mL/min/1.73 m^2^ (CKD stage G1-G3a), (2) baseline eGFR ≥ 60 mL/min/1.73 m^2^ (CKD stage G1-G2), and (3) baseline eGFR between 20 and 30 mL/min/1.73 m^2^.

## Results

### Baseline characteristics

Some participating centers were unable to collect the true endpoints of death and dialysis initiation; thus, participants from these centers were excluded. Therefore, although 152,815 participants were registered in the database, the number included in this study was 31,616. Among them, participants that did not have multiple eGFR measurements needed to calculate eGFR slope (18,713 for 1-year slope, 19,972 for 2-year slope, and 22,293 for 3-year slope), participants with missing covariates (1575 for hemoglobin, 2356 for serum albumin, and 6930 for serum CRP), and participants with a baseline eGFR less than 30 ml/min/1.73 m^2^ (2793) were excluded. The number of participants analyzed to calculate the eGFR slope for 1–3 years were 7768, 6778, and 5219, respectively. Table 1 presents the participants’ background information. The average observation periods of 1-year slope, 2-year slope, and 3-year slope were 877 ± 491 days, 706 ± 346 days, and 495 ± 215 days. The number of deaths during the observation period was 827 (10.7%), 533 (7.9%), and 317 (6.1%), respectively, whereas the incidence of dialysis initiation was very low, with 28 (0.4%), 24 (0.4%), and 14 (0.3%) participants, respectively. The incidences of CKD stage G5 were 186 (2.4%), 129 (1.9%), and 71 (1.4%), respectively. When the baseline eGFR was limited to participants with 60 ml/min/1.73 m^2^ or higher (CKD stages G1-G2), the incidence of dialysis initiation was 6 (0.1%), 6 (0.1%), and 4 participants (0.1%), respectively. Even when limited to participants with 45 ml/min/1.73 m^2^ or higher (CKD stages 1-3a), the incidence of dialysis initiation was also low, with 11 (0.2%), 11 (0.2%), and 8 participants (0.2%), respectively. Therefore, it was impossible to conduct a subgroup analysis of dialysis initiation based on CKD stage. However, a subgroup analysis according to the CKD stage was performed to determine the incidence of CKD stage G5.

**Table 1:** Baseline characteristics and outcome events

	1-year slope	2-year slope	3-year slope
N	7768	6778	5219
Age	64.9 ± 15.5	64.0 ± 15.6	63.6 ± 15.2
Male (%)	4176 (53.8)	3618 (53.4)	2758 (52.9)
Diabetes mellitus (%)	1464 (18.9)	1171 (17.3)	868 (16.6)
Antihypertensive medication prescription(%)	2307 (30.0)	1901 (28.1)	1492 (28.6)
RASI prescription (%)	1343 (17.3)	1.117 (16.5)	897 (17.2)
eGFR (ml/min/1.73m^2^)	74.0 ± 25.3	74.9 ± 26.1	74.5 ± 25.2
Hemoglobin (g/dL)	13.2 ± 2.0	13.4 ± 1.9	13.5 ± 1.88
Serum albumin (mg/dL)	4.0 ± 0.5	4.0 ± 0.5	4.0 ± 0.5
C-reactive protein (mg/dL)	0.15 (0.06, 0.44)	0.13 (0.06, 0.36)	0.12 (0.05, 0.32)
Observation period (days)	877 ± 491	706 ± 346	495 ± 215
eGFR slope: least-squares method (ml/min/1.73m^2^/year)	+ 0.24 ± 18.62	− 3.00 ± 8.85	− 2.60 ± 8.05
eGFR slope: mixed-effects model (ml/min/1.73m^2^/year)	− 0.40 ± 10.25	− 3.14 ± 5.81	− 3.41 ± 4.26
All-cause mortality (/1000 person-years)	44.3	40.7	44.8
Dialysis initiation (/1000 person-years)	1.5	1.8	2.0
Incident of CKD stage G5 (/1000 person-years)	10.65	9.96	10.16

Values are presented as mean ± standard deviation or median (25th, 75th percentile) for continuous variables and as number and percentage for binary variables

*RASI* renin-angiotensin system inhibitor, *eGFR* estimated glomerular filtration rate, *CKD* chronic kidney disease

### eGFR slope and risk of ESKD

**Fig. 2:**
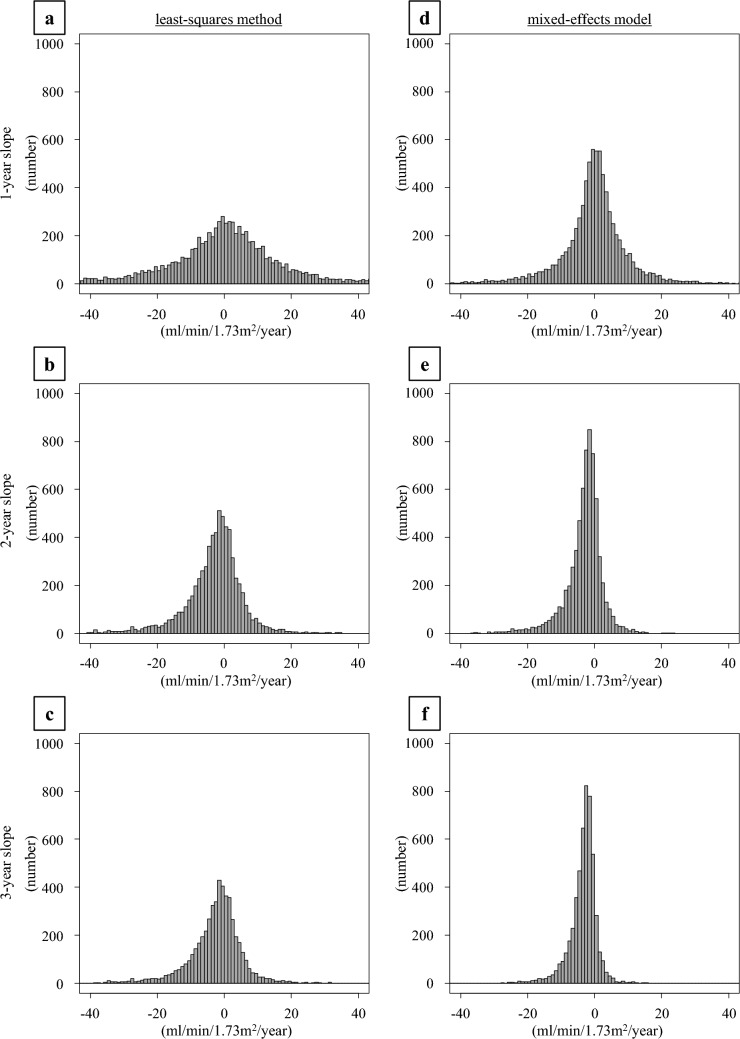
Distribution of slope values for each eGFR slope calculation period. The number of participants for each eGFR slope value is shown as a histogram. (**a**) 1-year slope calculated using the least-squares method, (**b**) 2-year slope calculated using the least-squares method, (**c**) 3-year slope calculated using the least-squares method, (**d**) 1-year slope calculated using the mixed-effects model, (**e**) 2-year slope calculated using the mixed-effects model, and (**f**) 3-year slope calculated using the mixed-effects model

**Fig. 3:**
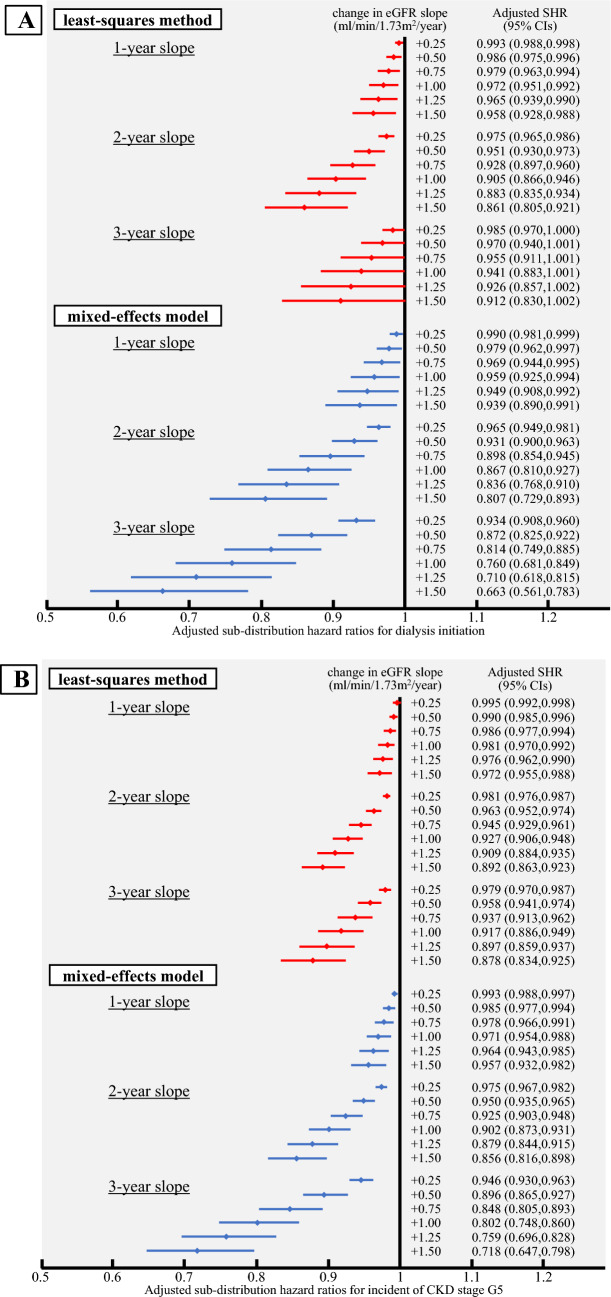
Adjusted sub-distribution hazard ratios for ESKD occurrence by change in eGFR slope. For each of the 1–3 year periods of the eGFR slope, the sub-distribution hazard ratios (SHRs) and 95% confidence intervals (CIs) for ESKD occurrence were shown for the range of change in eGFR slope of + 0.25 to + 1.50 ml/min/1.73 m^2^. (**a**) Adjusted SHRs for dialysis initiation and (**b**) Adjusted SHRs for the incident of CKD stage G5. The estimation of SHRs was performed using a Fine-Gray proportional hazards regression model, with death as a competing risk. The multivariate analysis was adjusted for age, sex, eGFR, hemoglobin, serum albumin, C-reactive protein, antihypertensive medication prescription, renin-angiotensin system inhibitor prescription, and diabetes mellitus

The distribution of the eGFR slope is shown in Fig. 2. In both the mixed effects model and least-squares method, the 1-year slope had a larger range of fluctuation than the 2- and 3-year slopes. Additionally, compared to the eGFR slope estimated using the mixed-effects model, the eGFR slope estimated using the least-squares method tended to have a larger range of fluctuation. Next, we examined the relationship between the eGFR slope and sub-distribution hazard ratio (SHR) for ESKD. For each calculation period of 1–3 years, we plotted the adjusted SHRs for ESKD as the initiation of dialysis (Fig. 3A) and as the incidence of CKD stage G5 (Fig. 3B) according to the magnitude of change in the eGFR slope. When ESKD was defined as initiation of dialysis, the adjusted SHRs corresponding to a gradual decrease in eGFR slope of + 0.5 to + 1.0 ml/min/1.73 m^2^/year were 0.986–0.972 for the 1-year slope estimated by the least squares method, 0.951–0.905 for the 2-year slope, and 0.970–0.941 for the 3-year slope. Similarly, for the 1-year slope estimated by the mixed-effects model, the adjusted SHRs were 0.979–0.959, 0.931–0.867 for the 2-year slope, and 0.872–0.760 for the 3-year slope (Fig. 3A). When ESKD was defined as the incidence of CKD stage G5, the adjusted SHRs corresponding to a gradual decrease in eGFR slope of + 0.5 to + 1.0 ml/min/1.73 m^2^/year were 0.990–0.981 for the 1-year slope estimated by the least squares method, 0.963–0.927 for the 2-year slope, and 0.958–0.917 for the 3-year slope. Similarly, for the 1-year slope estimated by the mixed-effects model, the adjusted SHRs were 0.985–0.971, 0.950–0.902 for the 2-year slope, and 0.896–0.802 for the 3-year slope (Fig. 3B). A decrease in the SHRs was observed in the eGFR slope estimated by the mixed-effects model, and a tendency for the SHRs to further decrease in the SHR was observed as the calculation period of the eGFR slope increased. Similarly, the eGFR slope estimated by the least-squares method showed a larger decrease in SHRs for a calculation period of 2–3 years compared to 1 year. In terms of the SHRs for ESKD, defined as the initiation of dialysis, the 2-year slope showed a slightly greater decrease than the 3-year slope, which was different from the results obtained using the mixed-effects model.

### Subgroup analysis

As a subgroup analysis, Table 2 shows the adjusted SHRs for changes in eGFR slope according to CKD stage 1-3a (eGFR ≥ 45 ml/min/1.73 m^2^) or G1-G2 (eGFR ≥ 60 ml/min/1.73 m^2^), with ESKD as the outcome for the incidence of CKD stage G5. In the mixed-effects model, a slightly smaller decrease in the SHR was observed when targeting CKD with eGFR ≥ 60 ml/min/1.73 m^2^, and a greater decrease in the SHR was observed for the 3-year slope compared to the 2-year slope. There were no significant differences in the SHRs between the subgroups or in the eGFR slope calculation period using the least-squares method. In the population with more advanced renal failure, baseline eGFR between 20 and 30 ml/min/1.73m^2^, we analyzed the association between 2-year slope and incidence of CKD stage G5. Compared to the population with baseline eGFR ≥ 30 ml/min/1.73m^2^, there was a greater decrease in the SHR in the subgroup with baseline eGFR between 20 and 30 ml/min/1.73m^2^ (Supplementary Table S1).

**Table 2:** Adjusted sub-distribution hazard ratios for ESKD (incidence of CKD stage G5) by change in eGFR slope in eGFR subgroups

		Adjusted sub-distribution hazard ratios for incidence of CKD stage G5 (95% confidence intervals)					
		eGFR ≥ 45 ml/min/1.73m^2^			eGFR ≥ 60 ml/min/1.73m^2^		
	Least-squares method	1-year slope	2-year slope	3-year slope	1-year slope	2-year slope	3-year slope
Change in eGFR slope (ml/min/1.73m^2^/year)	** + 0.25**	0.997 (0.994,1.000)	0.985 (0.977,0.992)	0.984 (0.972,0.996)	0.998 (0.995,1.002)	0.989 (0.980,0.998)	0.993 (0.978,1.008)
	** + 0.50**	0.994 (0.987,1.000)	0.970 (0.955,0.985)	0.968 (0.945,0.992)	0.997 (0.990,1.003)	0.977 (0.960,0.995)	0.985 (0.956,1.015)
	** + 0.75**	0.991 (0.981,1.000)	0.955 (0.933,0.977)	0.952 (0.919,0.987)	0.995 (0.985,1.005)	0.966 (0.940,0.993)	0.978 (0.935,1.023)
	** + 1.00**	0.988 (0.975,1.000)	0.940 (0.912,0.969)	0.937 (0.893,0.983)	0.993 (0.980,1.007)	0.955 (0.921,0.991)	0.971 (0.914,1.031)
	** + 1.25**	0.985 (0.969,1.001)	0.926 (0.891,0.962)	0.922 (0.868,0.979)	0.992 (0.975,1.008)	0.944 (0.902,0.989)	0.964 (0.894,1.039)
	** + 1.50**	0.981 (0.963,1.001)	0.911 (0.870,0.954)	0.907 (0.844,0.975)	0.990 (0.971,1.010)	0.934 (0.884,0.986)	0.956 (0.874,1.047)

	Mixed-effects model	1-year slope	2-year slope	3-year slope	1-year slope	2-year slope	3-year slope
Change in eGFR slope (ml/min/1.73m^2^/year)	** + 0.25**	0.996 (0.990,1.001)	0.979 (0.968,0.990)	0.956 (0.936,0.977)	0.998 (0.992,1.003)	0.984 (0.971,0.997)	0.965 (0.940,0.991)
	** + 0.50**	0.991 (0.981,1.001)	0.958 (0.937,0.980)	0.914 (0.875,0.954)	0.995 (0.984,1.006)	0.968 (0.943,0.994)	0.932 (0.884,0.983)
	** + 0.75**	0.987 (0.971,1.002)	0.938 (0.907,0.971)	0.874 (0.819,0.932)	0.993 (0.976,1.009)	0.953 (0.915,0.991)	0.900 (0.831,0.974)
	** + 1.00**	0.982 (0.962,1.003)	0.919 (0.878,0.961)	0.835 (0.766,0.910)	0.990 (0.969,1.012)	0.937 (0.889,0.989)	0.869 (0.781,0.966)
	** + 1.25**	0.978 (0.953,1.003)	0.899 (0.850,0.951)	0.798 (0.717,0.889)	0.988 (0.961,1.015)	0.922 (0.863,0.986)	0.839 (0.735,0.957)
	** + 1.50**	0.973 (0.943,1.004)	0.880 (0.823,0.942)	0.763 (0.671,0.868)	0.985 (0.953,1.018)	0.907 (0.838,0.983)	0.810 (0.691,0.949)

Adjusted sub-distribution hazard ratios for incidence of CKD stage G5 by changes in eGFR slope for each eGFR slope period (1–3 years) in subgroups with eGFR ≥ 45 ml/min/1.73 m^2^ and eGFR ≥ 60 ml/min/1.73 m^2^ were shown. The estimation of SHRs was performed using a Fine-Gray proportional hazards regression model, with death as a competing risk. The multivariate analysis was adjusted for age, sex, eGFR, hemoglobin, serum albumin, C-reactive protein, antihypertensive medication prescription, renin-angiotensin system inhibitor prescription, and diabetes mellitus

## Discussion

In this study, we focused on Japanese patients with early CKD and examined the relationship between the eGFR slope and SHR of ESKD. As the eGFR slope decreased, a tendency toward a lower risk of ESKD was observed. It has also been suggested that using eGFR values over a period of 2 or 3 years is more appropriate when calculating the eGFR slope.

The calculation periods for the eGFR slope in this study were 1, 2, and 3 years. As shown in Fig. 2, when calculating the slope from 1-year eGFR values, larger fluctuations were observed in many participants than when using data from two or three years. This may be because when the calculation period of the slope is short, the number of eGFR measurements decreases, making it more susceptible to the effects of short-term measurement variations as well as the effects of changes in acute kidney injury and muscle mass. The NKF-FDA-EMA workshop also recommended using data from a follow-up period of 2 to 3 years for reliable calculation of the eGFR slope [8], and based on the results of our study, using the eGFR slope based on 2 or 3 years of eGFR was considered more appropriate than 1 year (Figs. 2 and 3). However, the optimal calculation period for the eGFR slope may differ depending on the characteristics of the study population. It is necessary to set the calculation period for the eGFR slope, considering factors such as underlying CKD and the baseline GFR. In intervention studies that produce acute effects, such as the initial dip in eGFR caused by SGLT2 inhibitors, it may not be possible to anticipate the existence or magnitude of such effects beforehand, and it would be appropriate to evaluate them using the total slope calculated from the start of the intervention. However, if the calculation period is short, there is a risk of underestimating the treatment effect due to the acute effect, and it is considered desirable to have an observation period of at least 2–3 years. Therefore, further research is needed to address the challenge of determining whether a period of two years, three years, or longer is optimal.

Next, in the primary analysis, where ESKD was defined as the initiation of dialysis, there was a tendency for the SHR to decrease as the amount of change increased in the 3-year slope compared to the 2-year slope calculated by the mixed-effects model, while no such tendency was observed in the eGFR slope calculated by the least squares method. In the secondary analysis, in which ESKD was defined as the incidence of CKD stage G5, both the mixed effects model and the least squares method showed a greater decrease in the SHR in the 3-year slope than in the 2-year slope (Fig. 3). This is thought to be because the variance was smaller in the mixed-effects model owing to correction by estimated values, whereas the variability increased in the least-squares method owing to the influence of the fluctuation of eGFR values in individual participants. Furthermore, because the variability of the eGFR slope may have influenced the estimation of the SHR, there may have been differences in the SHRs obtained from each model. In estimating SHR, the variability of the eGFR slope may have affected the results, leading to differences between the two models. Regarding which method is more appropriate, it depends on the nature of the data to be analyzed and the purpose of the study. Another possible factor is that the incidence of dialysis initiation was relatively low, at approximately 0.2–0.4%, compared with the incidence of CKD stage G5 (approximately 1.4–2.4%). This may have resulted in greater variability in the SHR estimates in both models, leading to a decrease in estimation accuracy. In particular, the 3-year eGFR slope had a shorter observation period mean of 495 ± 215 days, compared to the 1-year slope (877 ± 491 days) and the 2-year slope (706 ± 346 days), which may also have had an impact.

We compared the results of this study with previous studies [8, 9]. The NKF-FDA-EMA workshop suggested that eGFR slope reduction 0.5–1.0 ml/min/1.73 m^2^/year may be associated with lower ESKD risk. In this study, we observed a trend towards a lower ESKD risk as the 2-year and 3-year slopes became more gradual. This trend was particularly strong for the 3-year slope and was consistent with the finding that a gradual decrease in the eGFR slope was associated with lower ESKD risk, as suggested by the NKF-FDA-EMA workshop. However, considering the limitations discussed later, such as the likelihood of worse renal outcomes in the population used in this study compared with the general CKD population, further investigations, including verification in multiple cohorts, are necessary to extrapolate the results of the NKF-FDA-EMA workshop to the general Japanese population with early CKD. Moreover, in the latest study, it has been reported that combining changes in albuminuria and the slope of eGFR improves the accuracy of endpoint estimation [16], indicating the potential for further research in this field.

The limitations of this study include, first, that it was an observational study using a database based on electronic medical record information, and unmeasured confounding factors could not be considered. For example, information such as a history of cardiovascular disease, blood pressure, smoking history, and physical activity, which are thought to be closely related to renal prognosis, was not collected from the database. Additionally, factors related to CKD progression, such as albuminuria or proteinuria, were often missing and were not adjusted for as covariates. Therefore, there is a possibility that the confounding adjustments were insufficient. Second, in this study, only data from facilities in the J-CKD-DB-Ex that included information on mortality and dialysis initiation were analyzed; therefore, the external validity of the analysis results was limited. Third, the data period of the J-CKD-DB-Ex was a maximum of 5 years, and there were few participants of dialysis initiation during the observation period, except for the eGFR slope calculation period. Particularly, when limited to participants with a baseline eGFR of > 45 ml/min/1.73 m^2^ or > 60 ml/min/1.73 m^2^, the number of outcomes was rare. Further investigations are needed to determine whether similar results can be obtained when limited to an earlier CKD population. Fourth, this study had multiple selection biases. The J-CKD-DB-Ex targets university hospitals in Japan and is considered to have a higher severity of CKD than the general CKD population. Therefore, the renal prognosis of the analyzed population is expected to be worse than that of the general Japanese population with CKD. In addition, there was selection bias associated with the criteria for registering patients in the J-CKD-DB-Ex. The inclusion criteria of the J-CKD-DB were as follows: (1) age ≥ 18 years old and (2) proteinuria ≥ 1 + (dipstick test) and/or eGFR < 60 mL/min/1.73 m^2^ at any point during the study period. Therefore, only patients who developed eGFR of < 60 ml/min/1.73 m^2^ or proteinuria ≥ 1 + would be selected among participants with a baseline eGFR of 60 ml/min/1.73 m^2^ or higher, resulting in an inevitably worse renal prognosis population. Regarding this, we compared the mean eGFR slope in each group of the study by Grams et al. [9], which was cited in the NKF-FDA-EMA workshop, with that of our study. According to this, the rate of renal function decline at baseline in the participants analyzed in this study did not deviate significantly from that reported in previous studies.

## Conclusion

Using the J-CKD-DB-Ex, a real-world database of Japanese patients with CKD, we investigated whether the relationship between a slower eGFR slope and a decreased risk of ESKD, as presented in the NKF-FDA-EMA workshop, also applies to patients with early CKD in Japan. While the generalizability of this study has several limitations, it suggests that a slower eGFR slope calculated from eGFR values over two or three years is associated with a decreased risk of ESKD.

## Supplementary Information

Below is the link to the electronic supplementary material.

Supplementary file1 (PPTX 67 KB)

## Data Availability

The datasets were generated at Kawasaki Medical School. Derived data supporting the findings of this study are available from the corresponding author on reasonable request.
