# Supplementary material for: eGFR slope as a surrogate endpoint for clinical study in early stage of chronic kidney disease: from The Japan Chronic Kidney Disease Database
Source: Clin Exp Nephrol. 2023 Jul 19;27(10):847–56. doi: 10.1007/s10157-023-02376-4 (PMC10504220; doi:10.1007/s10157-023-02376-4)
Supplement: Supplementary file 1 — Supplementary file1 (PPTX 67 KB) [file 10157_2023_2376_MOESM1_ESM.pptx]

## Slide 1
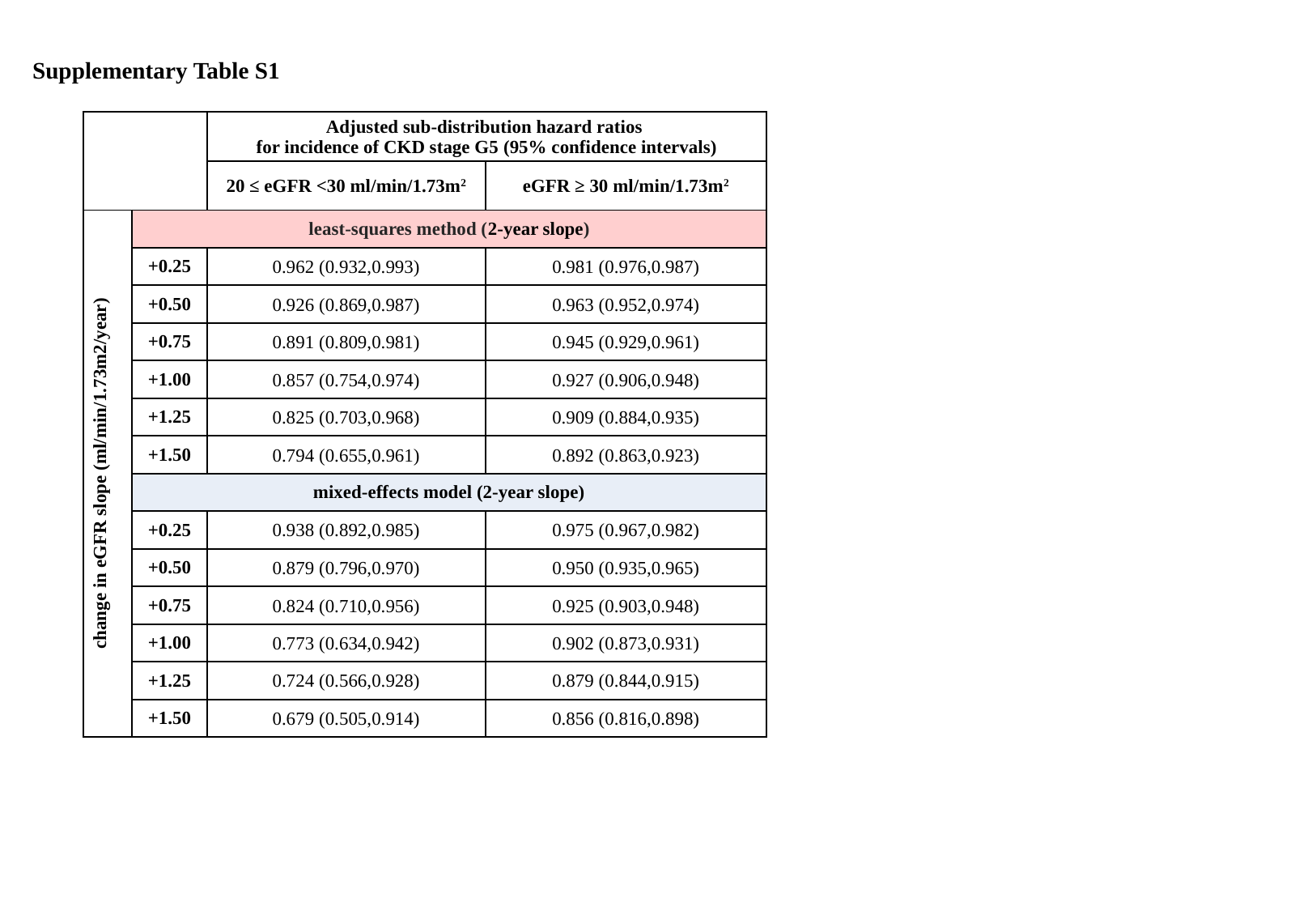

Supplementary Table S1
| | | Adjusted sub-distribution hazard ratios for incidence of CKD stage G5 (95% confidence intervals) | |
| --- | --- | --- | --- |
| | | 20 ≤ eGFR <30 ml/min/1.73m2 | eGFR ≥ 30 ml/min/1.73m2 |
| change in eGFR slope (ml/min/1.73m2/year) | least-squares method (2-year slope) | | |
| eGFR slope reduction (ml/min/1.73m2/year) | +0.25 | 0.962 (0.932,0.993) | 0.981 (0.976,0.987) |
| | +0.50 | 0.926 (0.869,0.987) | 0.963 (0.952,0.974) |
| | +0.75 | 0.891 (0.809,0.981) | 0.945 (0.929,0.961) |
| | +1.00 | 0.857 (0.754,0.974) | 0.927 (0.906,0.948) |
| | +1.25 | 0.825 (0.703,0.968) | 0.909 (0.884,0.935) |
| | +1.50 | 0.794 (0.655,0.961) | 0.892 (0.863,0.923) |
| | mixed-effects model (2-year slope) | | |
| | +0.25 | 0.938 (0.892,0.985) | 0.975 (0.967,0.982) |
| | +0.50 | 0.879 (0.796,0.970) | 0.950 (0.935,0.965) |
| | +0.75 | 0.824 (0.710,0.956) | 0.925 (0.903,0.948) |
| | +1.00 | 0.773 (0.634,0.942) | 0.902 (0.873,0.931) |
| | +1.25 | 0.724 (0.566,0.928) | 0.879 (0.844,0.915) |
| | +1.50 | 0.679 (0.505,0.914) | 0.856 (0.816,0.898) |
